# Role of *Kader Siaga Bencana* in the flood management of selected *Kampung Siaga Bencana*

**DOI:** 10.4102/jamba.v16i1.1645

**Published:** 2024-07-31

**Authors:** Fatmah Fatmah, Rachmadhi Purwana, Mizan F. Bisri

**Affiliations:** 1 Disaster Management Program, School of Environmental Science, University of Indonesia, Jakarta, Indonesia; 2 Graduate School of International Cooperation Studies, Kobe University, Kobe, Japan

**Keywords:** flood management, *posyandu cadres*, disaster preparedness cadres, disaster alert villages, knowledge, practice

## Abstract

Health cadres and community leaders’ empowerment to become flood disaster preparedness cadres (*Kader Siaga Bencana,* KSB) still needs to be improved. The study aimed to assess flood management practices among families with disaster-vulnerable groups, as well as KSB candidates (*posyandu*/health cadres, community leaders) in flood management initiatives in flood-prone areas. The study used a cross-sectional design of 100 families with flood-prone groups and 100 health cadres and community leaders. They were selected using simple random sampling in five selected villages of Jakarta city affected by floods and Disaster Alert Villages (*Kasmpung Siaga Bencana,* DAV). Most family respondents had a low knowledge level of DAV, and inadequate knowledge of flood warning system, emergency response plan, and flood management practices, despite having good family support. Health cadres and community leaders as KSB candidates were assessed using four parameters of knowledge on KSB – flood early warning system, emergency response plan, natural hazards and flood knowledge, and flood management practice. With their sound understanding of natural hazards and floods, health cadres and community leaders were 4.52 times more likely to have good flood management practices compared to those with inadequate knowledge. Both elements of the community have the potential to be KSB because of good disaster knowledge and the presence of the flood early warning system in the community. Intervention studies to increase the knowledge and practical ability of KSB in disaster management are also needed.

**Contribution:**

The research assessed the initial readiness of health cadres and community leaders to become disaster preparedness cadres in flood management. It is necessary to provide training to develop their expertise as KSB.

## Introduction

Flooding is one of the most common natural hazards, with particularly destructive effects in low-income countries. More than 90% of the developing countries addressed frequent floods in their reports. In 2011, severe floods were reported in Mozambique, Namibia, South Africa, and Uganda in Africa; Brazil, Colombia, and Mexico in South America, and Cambodia, China, India, Korea, Pakistan, the Philippines, and Thailand in Asia. There were over 1000 people killed in the

Philippines and Colombia caused by causing significant material damage, particularly in the above-mentioned developed countries (Kundzewicz et al. 2005).

The occurrence of flood disasters are common in Indonesia because it has a tropical climate with two seasons: the dry season and the rainy season, characterized by extreme weather changes, temperature, and wind direction (Chan et al. 2016). These extreme changes affect humans negatively through the occurrence of hydrometeorological disasters such as floods, landslides, forest fires, and droughts. From January to October 2023, there have been 3056 disasters, with floods (893 cases) and extreme weather (861 cases) being the most common (Annur 2023). Because of its location on the northwest coast of Java Island, as well as the fact that DKI Jakarta Province, which is also the capital of Indonesia, is home to the mouth of the Ciliwung River in Jakarta Bay, the province becomes a disaster-critical or disaster-prone area, particularly on account of floods. The average elevation of the area in Jakarta is 8 m above sea level. Climate change and Jakarta’s rapidly developing urban areas have contributed significantly to the frequency of floods in this province. Almost 17% of the total land area of Jakarta is predicted to be submerged by 2030, and most of the areas of Jakarta are under the threat of sinking by 2050 (Kulp & Benjamin 2019).

Natural hazards caused by climate change affect older people to a greater extent. It’s essential to understand how aware and prepared they are to deal with disasters. Several studies have indicated that older people may be less aware of and less prepared for disasters than the general population. However, it has been found that Canadian older people’s perceptions of risk are often associated with specific risks related to their geographic location (Bogdan et al. 2024). A significant proportion of older individuals residing in flood-prone areas of Bangkok have never participated in community-based flood preparedness and management training programs (Sawangnate, Benjawan & Suthirat 2023). Hence, it is imperative to increase awareness of flood preparedness among older people, one of the vulnerable groups inordinately affected by natural hazards.

One of the promotive and preventive actions that can be carried out comprehensively and collaboratively across sectors to minimize the impact of flooding on older people is to have Disaster Preparedness Cadres (*Kader Siaga Bencana,* KSB). These cadres are the community groups that improve the community’s disaster preparedness, prevention, and mitigation, and play an important role in quick responses to health issues. Although these cadres have not been officially included in Indonesia’s disaster emergency management plans, there is evidence that they are indeed involved in disaster responses in Indonesia, illustrating their crucial role in disaster management in society. Therefore, it is essential to improve disaster information literacy and skills related to disaster preparedness and flood management among cadres for flood-prone groups in urban areas. This includes pre-disaster mitigation and preparedness, emergency response during floods, and post-disaster recovery. Cadres are selected as the community group studied in this study because they are at the forefront of disaster management and can transfer disaster management knowledge to disaster-prone communities (Gustina 2020).

Cadre empowerment is one of the principles of disaster management under the Community-Based Disaster Management (CBDM) approach. This approach encourages grassroots communities to manage local disaster risks, helping the community to organize themselves to be self-sufficient in dealing with disasters before, during, and after disasters. The CBDM includes community activities such as disaster simulation, training, community-based disaster risk reduction education, and training on Basic Living Assistance (BHD) as the first steps of emergency relief (Herianto, Nulhaqim & Rachim 2015). Several studies in Indonesia have elaborated the role of cadres in disaster emergency responses to minimize the impact of the disaster on the victims, facilitate coordination, make reporting and coordination easier at the village and sub-district levels, and cadres’ quick responses in the event of a disaster (Maharani 2021; Puspitasari, Depi & Rismayanti 2021; Tiffany & Clarita 2021; Ulfa & Azizah 2021; Zuliani & Suhendi 2021). *Kader Siaga Bencana* is a Safe Community organization that supports the achievement of safe and healthy conditions in the community through the active roles played by community groups, including the private sector and professionals, which work in synergy with the government in emergency responses and disaster management. The essence of *Safe Community* is efforts by the community, from the community, and for the community, under the government’s support as a facilitator towards creating healthy and safe conditions (Zuliani & Suhendi 2021). Studies on KSB’s role in disaster prevention in DKI Jakarta Province have never been conducted despite their significant role they play in reducing flood risk, especially for flood-vulnerable groups. Thus, a study that assesses the knowledge and practice of families with group(s) vulnerable to disasters and cadres of integrated health posts [*Pos Pelayanan Terpadu, posyandu*] in urban areas of DKI Jakarta regarding disaster management is essential before preparing for the establishment of KSB in the future. This study aimed to assess the knowledge and practice of flood management among families with groups vulnerable to flood, health cadres, religious leaders, and community leaders in the selected flood-prone areas of Jakarta city.

## Research methods and design

### Study design

This cross-sectional study was conducted among 200 respondents living in the five selected villages of Rawa Buaya (West Jakarta), South Pengadegan (South Jakarta), Cawang (East Jakarta), Karet Tengsin (Central Jakarta), and Pejagalan (North Jakarta) in August 2023. These five villages were selected because they were among the 25 flood-prone villages in the province in 2023 (Azzahra 2023).

### Population and sample

The study population comprised the entire population of Rawa Buaya (West Jakarta), South Pengadegan (South Jakarta), Cawang (East Jakarta), Karet Tengsin (Central Jakarta), and Pejagalan (North Jakarta) villages, with a high Flood Risk Index of 21.03 in 2023 (National Board for Disaster Management [BNPB] 2021). The inclusion criteria for this study were people who had experienced flooding both inside and outside the home for at least the last 3 years and who had at least one family group that was categorized as one of the vulnerable groups (infant, toddler, child, pregnant woman, nursing mother, older people, and person with disability), a cadre of *posyandu* for under-five children, a cadre of *posbindu* for older people, and a local community/religious leader. The sampling technique was performed using the simple random sampling from the neighbourhoods (Rukun Warga, RW) affected by floods, at least in 2020. A total of 100 respondents from families with groups vulnerable to flood and 100 respondents from cadres from *posyandu* for under-five children, cadres for *posbindu* for older people, and local community/religious leaders participated in this study. The sample size was estimated using Slovin’s formula to get a representative sample of respondents (Jerri & Joyce 2012). Slovin’s formula estimated the sample size to be 100 individuals for each type of respondent: families with flood-prone groups and health cadres and community leaders.

$$n=\frac{N}{\left(1+\text{N}{\text{.e}}^{2}\right)}$$

The sample size, *N* is the total population affected by the flood at five selected villages (250 336 people) and e refers to the error margin at 10%.

### Study location

**FIGURE 1:**
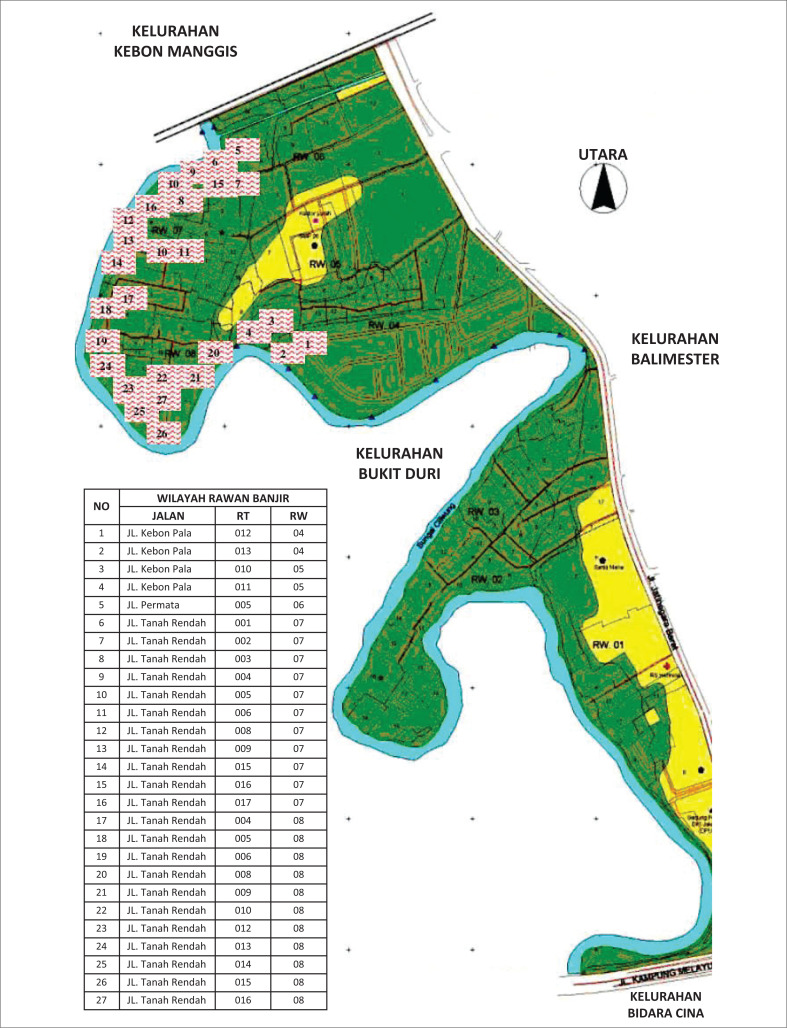
Flood hazard map of Kampung Melayu Urban Village.

The study was conducted in the five flood-prone villages in DKI Jakarta Province: Rawa Buaya (West Jakarta), South Pengadegan (South Jakarta), Kampung Melayu (East Jakarta), Pejagalan (North Jakarta), and Karet Tengsin (Central Jakarta). These villages had been established as Disaster Alert Villages (Kampung Siaga Bencana, KSB). Kampung Melayu Village is one of the villages on the banks of the Ciliwung River. It has been partially evicted and relocated to West Jatinegara because of the normalization of the Ciliwung River (Figure 1). Nearly 920 households in Kampung Melayu have been evicted because of this program (Karima, Purwantiasning & Prayodi 2018).

**FIGURE 2:**
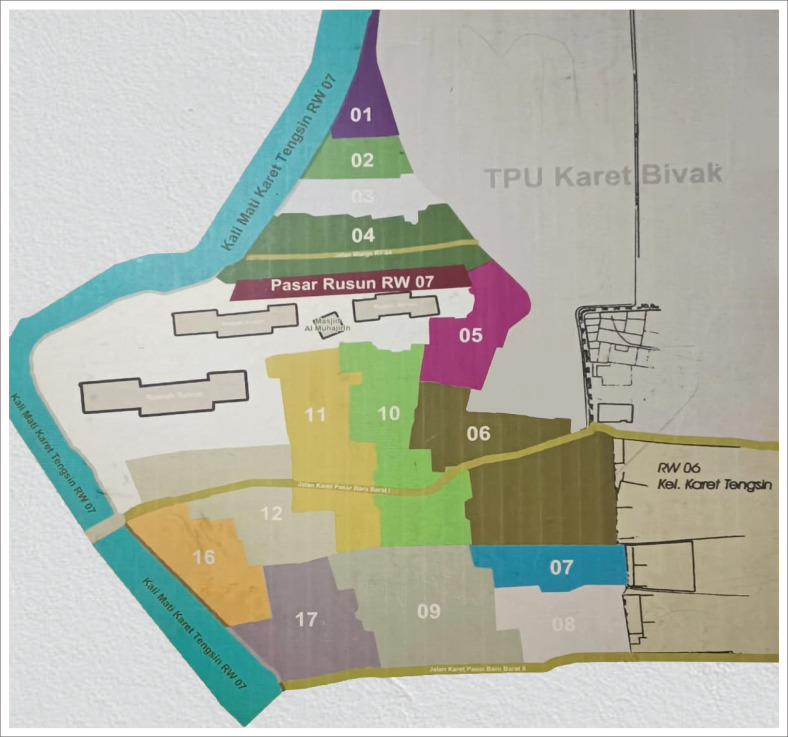
Flood hazard map of Karet Tengsin Urban Village.

**FIGURE 3:**
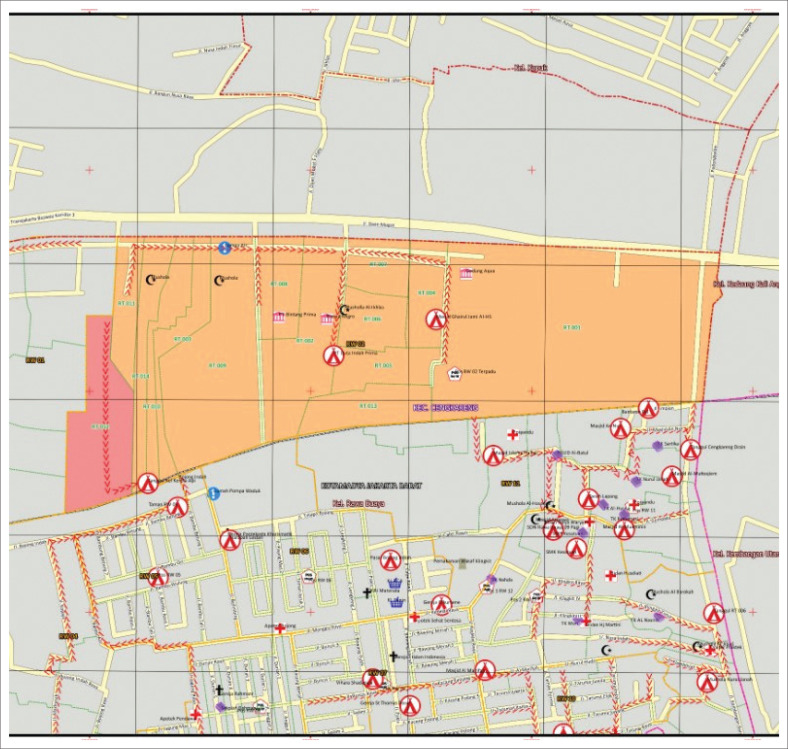
Flood hazard map of Rawa Buaya Urban Village.

**FIGURE 4:**
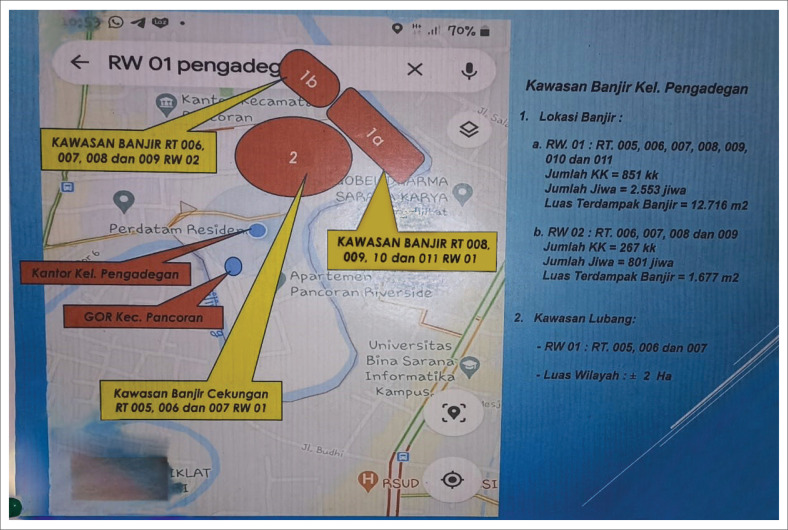
Flood hazard map of South Pengadegan Urban Village.

**FIGURE 5:**
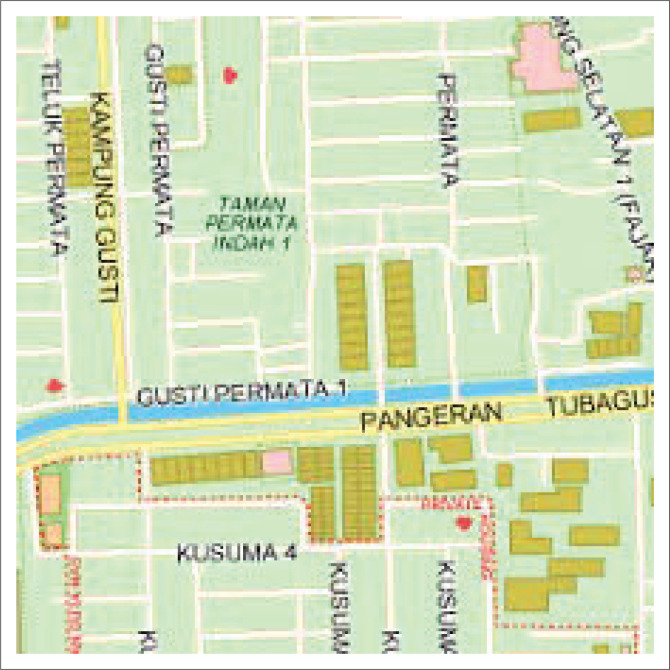
Flood hazard map of Pejagalan Urban Village.

Karet Tengsin Village is situated on a relatively flat terrain and is flanked by two rivers: the West Flood Canal and the Krukut River. These two rivers make the village susceptible to flooding, particularly during severe floods every 5 years. The densely populated settlement in this area is located along the Krukut Riverbank: a low plateau that can potentially be affected by flooding when there is an overflow of river water (Figure 2). Rawa Buaya Village is located in an area surrounded by three streams: Mookevart River, Anak Kali Angke, and Sanggrahan River (Figure 3). The region is dominated by housing and industrial estates, which has reduced the catchment area. The causes of flooding in this area include poorly organized water and drainage systems, cut drainage channels, narrowing and silting of river bodies, and damage to water pumps. Additionally, reservoirs do not function properly because of silt and garbage deposits (Putro 2010). Pengadegan Village is located on the bank of the Ciliwung River, which makes it vulnerable to flooding. Lubang Kampong, situated in a lowland area below the river surface, is also at risk. The flooding affects around 3 hectares of RW 01 and RW 02 (Figure 4). Pejagalan Village is another flood-prone area because of its proximity to the Angke River (Figure 5).

### Study framework

This study applied the flood disaster management framework through an assessment of socio-demographic characteristics (marital status, age, final education, employment, number of people living in the house, number of biological children, total income, and size or number of family groups); knowledge of the Disaster Preparedness Village (definition, tasks); knowledge of natural hazards and floods (definitions, types of natural hazards, causes, impacts, groups prone to natural hazards, need for disaster-prone groups to prepare for floods); family practices in flood management (before, during, and after disaster); disaster warning systems (sources of information, efforts made when hearing information about floods); support for families with groups vulnerable to flood (forms of support and assistance provided); and emergency response plan parameters (division of tasks in self-rescue actions during floods; self-evacuation during floods; availability of a map, place, route, and meeting point for family evacuation and shelter in the event of flooding; relatives or family groups who provide temporary shelter during floods; availability of first-aid box or essential medicines for first aid during floods; self-rescue initial measures in the event of flood; family groups who had received training on first aid and flood evacuation; the presence or absence of evacuation routes during floods; flood emergency preparation; knowledge on whether or not evacuation routes are available; availability of family communication equipment [cell phone or radio or Handy Talky] during flooding; availability of family lighting equipment for flood emergency [flashlight or emergency lamp or generator]; the availability of disaster preparedness bags; owning essential numbers that can be contacted in an emergency [hospital, police, fire department]; and whether or not to get flood preparedness education and materials).

### Data analysis

Univariate analysis of socio-demographic characteristics (gender, marital status, age, late education, employment, number of people living in the house, number of biological children, total income, and size or number of family groups) was presented in the frequency distribution table. Knowledge of KSB, natural hazards, and flood, as well as the emergency response plan parameters and flood management practices (before the disaster, during emergency response, and after the disaster), were displayed in the +standard deviation (s.d.) mean and frequency distribution. Bivariate analysis was used to assess differences in flood management practices (pre, during, and post) in two categories of inadequate and reasonable based on the socio-demographic characteristics, knowledge of KSB, understanding of natural hazards and floods, flood warning systems, and emergency response plan parameters by applying the Chi-Square test with a significance level of *p* < 0.05. Multiple logistics multivariate analysis was used to assess the determinant independent variables that determine the practices of families with groups vulnerable to flood, health cadres ([*posyandu/posbindu*] of older people or mosquito larva monitoring [*jumantik*], and community leaders in flood management).

### Ethical considerations

An application for full ethical approval was made to the Ethics Commission for Health Research and Development (KEPPK) of the Sint Carolus School of Health Sciences and ethics consent was received on 20 July 2023. The ethics approval number is 112/KEPPKSTIKSC/VII/2023.

## Results

### Families with groups vulnerable to flood

The socio-demographic characteristics of families with groups belonging to one of the vulnerable groups to flood are presented in Table 1 (gender, marital status, age group, education level, occupation, income, number of family groups living in the house, and number of biological children living in the house). Married women comprised of the majority of respondents. Most respondents were between the age of 20 and 34 years, with a mean age of 40.9 ± 15.5. Most had at least a high school education, worked informally, and had a monthly income of 1–3 million. Living with a husband, children, and other family groups is the respondents’ most common living arrangement. Most respondents had 1–2 biological children. Most respondents had a low level of knowledge about KSB, inadequate flood warning system and emergency response plan parameters, and inadequate flood disaster management practices, yet they had good family support (Table 2).

**TABLE 1:** Sociodemographic characteristic.

Variable	Number of participants (*n*)	Percentage (%)
**Sex**		
Male	39	39.0
Female	61	61.0
**Marital status**		
Single or widow or widower	25	25.0
Married	75	75.0
**Age group (in years)**		
20–34	43	43.0
35–49	30	30.0
> 49	27	27.0
**Final education**		
Graduated from junior high school or below	46	46.0
Graduated from senior high school or above	54	54.0
**Job status**		
Work irregularly	56	56.0
Work regularly	44	44.0
**Live at the same house with**		
Husband or wife or children and other family	55	55.0
Husband or wife or children	45	45.0
**Number of owned children (person)**		
> 2	45	45.0
1–2	55	55.0
**Monthly income (IDR)**		
< 1 million	23	23.0
1–3 million	45	45.0
> 3 million	32	32.0

Note: Age group mean is 41.0; standard deviation is 15.0. The minimum to maximum is 20.0–78.0.

IDR, income-driven repayment.

**TABLE 2:** Preparedness parameter and flood management practice of respondents with flood-prone group.

Variable (number of questions, value range)	Mean	Median	Min–Max	s.d.	Poor		Good	
					*n*	%	*n*	%
Knowledge of *Kampung Siaga Bencana* (7 items, 0–100)	19.0	0.0	0.0–86.0	26.0	66	66	34	34
Flood early warning system (12 items, 0–100)	30.0	25.0	0.0–75.0	17.0	51	51	49	49
Emergency response plan (15 items, 0–100)	56.0	53.0	13.3–100.0	13.0	54	54	45	45
Knowledge on natural hazard and flood (39 items, 0–100)	33.0	31.0	7.7–74.0	12.0	54	54	46	46
Family support (4 items, 0–100)	19.0	25.0	0.0–75.0	19.0	40	40	60	60
Practice before flood (18 items, 0–100)	31.0	29.0	5.6–72.0	13.0	58	58	42	42
Practice during flood (31 items, 0–100)	34.0	32.0	12.9–77.0	11.0	58	58	42	42
Practice after flood (8 items, (0–100)	31.0	25.0	0.0–100.0	19.0	60	60	40	40
Flood management practice (57 item, 0–100)	32.0	30.0	10.5–79.0	13.0	60	60	40	40

s.d., standard deviation.

Table 3 illustrates differences in pre-flood practices by age group, education, number of biological children, knowledge of natural hazards and floods, flood warning system, and family support. Flood response practices differ based on age group, education, number of biological children, income, knowledge of natural hazards and floods, flood warning system, family support, and emergency response plan parameters. Differences based on post-flood practices were found in variables such as age group, marital status, final education, employment status, income, knowledge of natural hazards and floods, and family support. Respondents’ flood management practices differed by age group, employment status, final education, knowledge of natural hazards and floods, flood warning system, and family support. Results of the multivariate analysis of logistic regression on practices before flood demonstrated that the availability of a good flood warning system would support good pre-flood practices by 6.36 times, compared to an inadequate flood warning system. A good flood warning system was 6.57 times more likely to support good flood response practices. A good level of knowledge of natural hazards and floods had a probability of good after-flood practices of 4.3 times higher compared to an inadequate level of knowledge of natural hazards and floods. A good flood warning system was 11.15 times more likely to lead to good flood management practices than an inadequate flood warning system (Table 4).

**TABLE 3a:** Differences in flood management practices based on socio-demographic characteristics, disaster knowledge, flood early warning systems, family support, and emergency response plans in families with members of flood-prone group.

Variable	Before flood				*p*	OR	95% CI OR	During flood				*p*	OR	95% CI OR
	Poor		Good					Poor		Good				
	*n*	%	*n*	%				*n*	%	*n*	%			
**Sex**														
Male	24	61.0	15	38.0	0.566	1.0	-	26	67.0	13	33.0	0.160	1.0	-
Female	34	56.0	27	44.0	-	1.0	1.0–4.0	32	52.0	29	47.0	-	2.0	0.8–4.0
**Marital status**														
Married	47	63.0	28	37.0	0.101	1.0	-	44	59.0	31	41.0	0.815	1.0	-
Single or widow or widower	11	44.0	14	56.0	-	2.0	1.0–5.0	14	56.0	11	44.0	-	1.0	0.4–3.0
**Age group (in years)**														
> 49	21	78.0	6	22.0	0.035	1.0	-	24	89.0	3	11.0	0.001	1.0	-
35–49	17	57.0	13	43.0	-	3.0	1.0–8.0	17	57.0	13	43.0	-	6.0	1.0–24.9
20–34	20	46.0	23	53.0	-	4.0	1.0–12.0	17	39.0	26	60.0	-	12.0	3.0–47.0
**Final education**														
Graduated from junior high school or below	33	72.0	13	28.0	0.010	1.0	-	38	83.0	8	17.0	0.001	1.0	-
Graduated from senior high school or above	25	46.0	29	54.0	-	3.0	1.0–7.0	20	37.0	34	63.0	-	8.0	3.0–21.0
**Working status**														
Irregularly work	36	64.0	20	36.0	0.151	1.0	-	36	64.0	20	36.0	0.151	1.0	-
Regularly work	22	50.0	22	50.0	-	2.0	1.8–4.0	22	50.0	22	50.0	-	2.0	0.8–4.0
**Live in the house with**														
Husband or wife or children, other family	31	56.0	24	44.0	0.714	1.0	-	31	56.0	24	44.0	0.714	1.0	-
Husband or wife or children	27	60.0	18	40.0	-	1.0	0.4–2.0	27	60.0	18	40.0	-	1.0	0.4–2.0
**Number of owned children (person)**														
> 2	31	69.0	14	31.0	0.046	1.0	-	31	69.0	14	31.0	0.046	1.0	-
1–2	27	49.0	28	51.0	-	2.0	1.0–5.0	27	49.0	28	51.0	-	2.0	1.0–5.0
**Monthly income (IDR)**														
< 1 million	15	65.0	8	35.0	0.725	1.0	-	17	74.0	6	26.0	0.013	1.0	-
1–3 million	25	56.0	20	44.0	-	1.0	0.5–4.0	29	64.0	16	36.0	-	2.0	0.5–5.0
> 3 million	18	56.0	14	44.0	-	1.0	0.5–4.0	12	37.0	20	62.0	-	5.0	1.0–15.0
**Knowledge on *Kampung Siaga Bencana***														
Poor	38	57.6	28	42.0	0.905	1.0	-	39	59.0	27	41.0	0.758	1.0	-
Good	20	58.8	14	41.0	-	1.0	0.4–2.0	19	56.0	15	44.0	-	1.0	0.5–3.0
**Knowledge on natural hazard and flood**														
Poor	39	72.2	15	28.0	0.002	1.0	-	43	80.0	11	20.0	0.001	1.0	-
Good	19	41.3	27	59.0	-	4.0	2.0–8.0	15	33.0	31	67.0	-	8.0	3.0–20.0
**Flood early warning system**														
Poor	37	72.5	14	27.0	0.003	1.0	-	37	72.0	14	27.0	0.003	1.0	-
Good	21	42.9	28	57.0	-	3.0	1.0–8.0	21	43.0	28	57.0	-	3.0	1.0–8.0
**Family support**														
Poor	31	77.5	9	22.0	0.001	1.0	-	33	82.0	7	17.0	0.001	1.0	-
Good	27	45.0	33	55.0	-	4.0	2.0–10.0	25	42.0	35	58.0	-	7.0	2.0–17.0
**Emergency response plan**														
Poor	32	61.5	20	38.0	0.456	1.0	-	37	71.0	15	29.0	0.006	1.0	-
Good	26	54.2	22	46.0	-	1.0	1.0–3.0	21	44.0	27	56.0	-	3.0	1.0–7.0

CI, confidence interval; OR, odd ratio; s.d., standard deviation.

**TABLE 3b:** Differences in flood management practices based on socio-demographic characteristics, disaster knowledge, flood early warning systems, family support, and emergency response plans in families with members of flood-prone group.

Variable	After flood				*p*	OR	95% CI OR	Flood management				*p*	OR	95% CI OR
	Poor		Good					Poor		Good				
	*n*	%	*n*	%				*n*	%	*n*	%			
**Sex**														
Male	22	56.0	17	43.0	0.947	1.0	-	25	64.1	14	36.0	0.503	1.0	-
Female	34	56.0	27	44.0	-	1.0	0.5–2.0	35	57.4	26	43.0	-	1.0	0.0 – 3.0
**Marital status**														
Married	46	61.0	29	39.0	0.063	1.0	-	12	48.0	13	52.0	0.157	1.0	-
Single or widow or widower	10	40.0	15	60.0	-	2.0	1.0–6.0	48	64.0	27	36.0		2.0	1.0 – 5.0
**Age group (in years)**														
> 49	21	78.0	6	22.0	0.013	1.0	-	24	88.9	3	11.0	0.001	1.0	-
35–49	17	57.0	13	43.0	-	3.8	1.0–8.0	18	60.0	12	40.0	-	5.0	1.0 – 22.0
20–34	18	42.0	25	58.0	-	5.0	2.0–14.0	18	41.9	25	58.0	-	1.0	3.0 – 43.0
**Final education**														
Graduated from junior high school or below	34	74.0	12	26.0	0.001	1.0	-	38	82.6	8	17.0	0.001	1.0	-
Graduated from senior high school or above	22	41.0	32	59.0	-	4.0	2.0–10.0	22	40.7	32	59.0	-	7.0	3.0 – 18.0
**Working status**														
Irregularly work	37	66.0	19	34.0	0.022	1.0	-	40	71.4	16	29.0	0.008	1.0	-
Regularly work	19	43.0	25	57.0	-	3.0	1.0–9.0	20	45.5	24	54.0	-	3.0	1.0 – 7.0
**Live in the house with**														
Husband or wife or children, other family	28	51.0	27	49.0	0.257	1.0	-	32	58.2	23	42.0	0.682	1.0	-
Husband or wife or children	28	62.0	17	38.0	-	1.0	0.3–1.0	28	62.2	17	38.0	-	0.8	0.4 – 2.0
**Number of owned children (person)**														
> 2	27	60.0	18	40.0	0.466	1.0	-	31	68.9	14	31.0	0.101	1.0	-
1–2	29	53.0	26	47.0	-	1.0	1.0 – 3.0	29	52.7	26	47.0	-	2.0	1.0 – 4.0
**Monthly income (IDR)**														
< 1 million	17	74.0	6	26.0	0.021	1.0	-	17	73.9	6	26.0	0.120	1.0	-
1–3 million	27	60.0	18	40.0	-	2.0	1.0 – 6.0	28	62.2	17	38.0	-	2.0	0.6 – 5.0
> 3 million	12	37.0	20	62.0	-	8.0	1.0–15.0	15	46.9	17	53.0	-	3. 0	1.0 – 10.0
**Knowledge on *Kampung Siaga Bencana***														
Poor	38	58.0	28	42.0	0.658	1.0	-	39	59,1	27	41.0	0.796	1.0	-
Good	18	53.0	16	47.0	-	1.0	0.5–3.0	21	61,8	13	38.0	-	1.0	0.4 – 2.0
**Knowledge on natural hazard and flood**														
Poor	39	72.0	15	28.0	0.001	1.0	-	44	81.5	10	18.0	0.001	1.0	-
Good	17	37.0	29	63.0	-	4.0	3.0–10.0	16	34.8	30	65.0	-	8.0	3.0 – 22.0
**Flood early warning system**														
Poor	32	63.0	19	37.0	0.166	1.0	-	38	74.5	13	25.0	0.003	1.0	-
Good	24	49.0	25	51.0	-	2.0	1.0–4.0	22	44.9	27	55.0	-	4.0	1.0 – 8.0
**Family support**														
Poor	29	72.0	11	27.0	0.007	1.0	-	34	85.0	6	15.0	0.001	1.0	-
Good	27	45.0	33	55.0	-	3.0	1.0 – 8.0	26	43.3	34	57.0	-	7.0	3.0 – 21.0
**Emergency response plan**														
Poor	33	63.0	19	36.0	0.118	1.0	-	36	69.2	16	31.0	0.050	1.0	-
Good	23	48.0	25	52.0	-	2.0	1.0 – 4.0	24	50.0	24	50.0	-	2.0	1.0 – 5.0

CI, confidence interval; OR, odd ratio; s.d., standard deviation.

**TABLE 4:** Multivariate analysis of the flood management practices dominant variable in families with flood-vulnerable members.

Variable	Logistic coefficients (B)	Standard error (s.e.)	Wald	db	*p*	OR	95% CI OR	
							BB	BA
**Before flood**								
Marital status *(Single or widow or widower vs. married)*	1.44	0.58	6.17	1	0.013	4.24	1.36	13.26
Flood early warning system *(Good vs. Poor)*	1.83	0.52	12.38	1	0.000	6.26	2.25	17.41
Family support before flood *(Good vs. Poor)*	1.75	0.52	11.45	1	0.001	5.77	2.09	15.92
Constant	−2.75	0.61	20.31	1	0.000	0.06	-	-
*-2LL=107.8, Cox and Snell R Square = 0.23, Nagelkerke R Square = 0.33, Omnibus Test Model = 28.285, p = 0.001*								
**During flood**								
Final education *(Graduated from senior high school or above vs. Graduated from junior high school or below)*	1.41	0.58	5.86	1	0.016	4.10	1.31	12.83
Knowledge on natural hazard and flood *(Good vs. Poor)*	1.73	0.61	8.04	1	0.005	5.64	1.71	18.68
Flood early warning system *(Good vs. Poor)*	1.88	0.59	10.04	1	0.002	6.57	2.05	21.05
Family support before flood *(Good vs. Poor)*	1.27	0.63	4.11	1	0.043	3.56	1.04	12.16
Constant	−3.82	0.77	24.75	1	0.000	0.02	-	-
*-2LL = 86.0, Cox and Snell R Square = 0.39, Nagelkerke R Square = 0.53, Omnibus Test Model = 50.05, p = 0.001*								
**After flood**								
Working status *(Regularly vs. Irregularly)*	0.89	0.44	4.05	1	0.044	2.43	1.02	5.79
Knowledge on natural hazard and flood *(Good vs. Poor)*	1.46	0.44	10.95	1	0.001	4.30	1.81	10.18
Constant	−1.34	0.38	12.78	1	0.000	0.26	-	-
*-2LL = 120.28 Cox and Snell R Square = 0.16, Nagelkerke R Square=0.21, Omnibus Test Model=16.9, p = 0.001*								
**Flood management**								
Working status *(Regularly vs. Irregularly)*	1.66	0,60	7.56	1	0.006	5.26	1.61	17.16
Knowledge on natural hazard and flood *(Good vs. Poor)*	2.15	0.62	11.95	1	0.001	8.59	2.54	29.10
Flood early warning system *(Good vs. Poor)*	2.41	0.66	13.34	1	0.000	11.15	3.06	40.69
Family support before flood *(Good vs. Poor)*	1.67	0.64	6.80	1	0.009	5.32	1.51	18.69
Constant	−4.53	0.87	26.86	1	0.000	0.01	-	-
*-2LL = 81.02 Cox and Snell R Square = 0.42, Nagelkerke R Square = 0.56, Omnibus Test Model = 53.59, p = 0.001*								

CI, confidence interval; OR, odd ratio.

### Cadres and community leaders

Most respondents have *dasawisma/posyandu/jumantik* cadre positions with equal gender proportion between males and females. The percentage of married respondents was almost five times higher than those who were not married (single, widow/widower). The majority of respondents were over the age of 50 years, with a mean of 46.9 years, and at least graduated from senior high school. Most respondents worked as *RT* (neighborhood association)*/RW* (citizen association) staff, volunteers, cadres, and community leaders. Respondents generally lived with their nuclear family, which consisted of the husband/wife and their children. Most respondents had 1–2 biological children with a monthly income of 1–3m (Table 5). The levels of knowledge of natural hazards and floods, flood warning systems, emergency response parameters, and pre-flood practices were classified as suitable for most respondents, except for knowledge of natural hazards and floods. The comparison between good flood management practices and after-flood practices was similar to inadequate practices (Table 6).

**TABLE 5:** Sociodemographic characteristic of health cadres and community leaders.

Variable	Number of participants (*n*)	Percentage (%)
**Position in the community**		
Chairman or staff of *RT or RW*	38	38.0
Youth organization or community leaders	23	23.0
*Dasawisma or posyandu* cadres	39	39.0
**Sex**		
Male	50	50.0
Female	50	50.0
**Marital status**		
Single or widow or widower	17	17.0
Married	83	83.0
**Age group (in years)**		
20–40	28	28.0
41–50	35	35.0
> 50	37	37.0
**Final education**		
Graduated from junior high school or below	33	33.0
Graduated from senior high school or above	67	67.0
**Working status**		
Irregularly work	44	44.0
Regularly work	35	35.0
Staf RW or RT or volunteer or health cadres or community leader	21	21.0
**Live in the same house with**		
Husband or wife or children with other family	41	41.0
Husband or wife or children with other	59	59.0
**Number of owned children**		
> 2	36	36.0
1–2	64	64.0
**Monthly income (IDR)**		
1–3 million	60	60.0
> 3 million	40	40.0

Note: Age group (in years) Mean is 47.0; standard deviation is 11.0; Min-Max is 19.0–70.0.

s.d., standard deviation; RT, neighborhood association; RW, citizens association; IDR, income-driven repayment.

**TABLE 6:** Preparedness parameter and flood management practice of health cadres and community leaders.

Variable (number of questions, value range)	Mean	Median	Min-Max	s.d.	Poor		Good	
					*n*	%	*n*	%
Knowledge of KSB (13 items, 0–100)	28.0	31.0	0.0–69.0	19.0	42	42	58	58
Food early warning system (12 items, 0–100)	34.0	33.0	0.0–92.0	17.0	39	39	61	61
Emergency response plan (15 items, 0–100)	70.0	73.0	47.0–93.0	11.0	49	49	51	51
Knowledge on natural disaster and flood (37 items, 0–100)	42.0	43.0	19.0–78.0	11.0	45	45	55	55
Practice before flood (15 items, 0–100)	44.0	47.0	13.0–87.0	14.0	49	49	51	51
Practice during flood (30 items, 0–100)	30.0	27.0	13.0–70.0	10.0	48	48	52	52
Practice after flood (9 items, 0–100)	37.0	35.0	11.0–67.0	15.0	50	50	50	50
Flood management practice (54 items, 0–100)	40.0	35.0	11.0–67.0	11.0	50	50	50	50

s.d., standard deviation.

Most respondents took several measures to protect residents with vulnerable family groups from floods. These measures included providing verbal explanations on self-evacuation steps, maps, and plans and disseminating and educating residents on flood control programs. Respondents mostly facilitated the self-evacuation of flood-prone groups to safer places during floods. In addition, almost all respondents prioritized rescuing children, pregnant women, nursing mothers, the elderly, and persons with disabilities during floods. This was because these groups had limited movement, could not help themselves, and were more susceptible to sickness. Post-flood respondents mainly focused on ensuring the health condition of flood-prone groups. However, providing nutritious food, medicine, and vitamins was the least frequently done effort by respondents after the flood (Table 7).

**TABLE 7:** Distribution of frequency of flood management practices, participation in disaster training, efforts to increase community preparedness, willingness to become KSB among health cadres and community leaders.

Variable	Number of participants (*n*)	Percentage (%)
**Before flood (*multiple answer*)**		
Explain verbally the steps/map/plan for self-evacuation	47	47
Practice self-evacuation plans/steps	26	26
Education and socialisation of flood prevention behaviour	50	50
**During flood (*multiple answer*)**		
Evacuate himself/herself via the evacuation route to a safe place	68	68
Prioritise residents vulnerable to flooding for evacuation	98	98
**After flood (*multiple answer*)**		
Ensure the health status is in good condition	65	65
Give the medicine and food if necessary	32	32
Give nutritious food and safe water	-	-
Efforts are made when hearing danger signs for groups vulnerable to flooding (*multiple answer*)	-	-
Help evacuate flood-prone communities to safe places	37	37
**Readiness becomes KSB (*single answer*):**		
Participation in the disaster training	-	-
Yes	42	42
No	58	58
**Type of disaster training attended before (*multiple answer*)**		
Disaster mitigation	34	34
Handling assistance to injured victims	10	10
Flood evacuation simulation	10	10
Basic Life Support (BLS)	4	4
**Readiness becomes KSB (*single answer*)**		
Yes	72	72
No	28	28
**The reasons for being willing to become a KSB (*single answer*)**		
Helping community	25	25
Participate in facing flood	50	50
A form of social activity in society	15	15
A form of social responsibility activity in society	10	10
**Willing to take part in KSB skills improvement training (*single answer*)**		
Yes	69	69
No	31	31

Almost half of all respondents had attended flood preparedness training held in the local village office, with most attending the training in 2018. Most training providers were from the DKI Jakarta Fire Brigade, Jakarta City of Regional Disaster Management Agency (*BPBD*), the local village government, and Wahana Visi NGO. The types of training attended were flood and earthquake evacuation simulations, first aid, injury management, and flood mitigation. Respondents made several efforts to improve community preparedness in their environment, including appeals not to litter carelessly, information sharing on self-evacuation during floods, community service practices, flood mitigation simulations, and information on drug preparation. When asked about their willingness to become KSB, most expressed their desire to join because it is a good cause to help residents and participate in managing floods. The KSB is also seen as a social cause for the community and a form of responsibility towards the community. Most respondents were willing to participate in disaster training to be more skilled in flood management and improve their knowledge (Table 7).

Flood management practices differ significantly based on knowledge of KSB, understanding of natural hazards and floods, flood warning systems, and emergency response plans (Table 8). The multivariate analysis showed that communities with a good flood early warning system were 6.33 times more likely to have pre-flood practices than communities with inadequate flood early warning systems. Variables significantly associated with flood emergency response practices were knowledge of KSB, understanding of natural hazards and floods, and emergency response plan parameters. The most dominant variable was knowledge of natural hazards and floods at 8.15. Understanding natural hazards and floods also dominated post-flood practices at 8.16 and flood management practices at 4.52 (Table 9).

**TABLE 8a:** Differences in flood management practices based on socio-demographic characteristics, *Kampung Siaga Bencana* knowledge, disaster knowledge, flood EWS, a demergency response plans for heat cadres and comm. leaders.

Variable	Before flood				*p*	OR	95% CI OR	During flood				*p*	OR	95% CI OR
	Poor		Good					Poor		Good				
	*n*	%	*n*	%				*n*	%	*n*	%			
**Position in the community**														
Chairman or staff of *RT* or *RW*	23	60.0	15	39.5	0.152	1.0	-	21	55.0	17	45.0	0.369	1.0	-
Youth organization or comm. leaders	11	48.0	12	52.0	-	2.0	1.0–5.0	9	39.0	14	61.0	-	2.0	1.0–5.0
Health cadres	15	38.0	24	61.0	-	2.0	1.0–6.0	22	56.0	17	44.0	-	1.0	0.4–2.0
**Sex**														
Male	28	56.0	22	44.0	0.161	1.0	-	27	54.0	23	46.0	0.689	1.0	-
Female	21	42.0	29	58.0	-	2.0	1.0–4.0	25	50.0	25	50.0	-	1.0	0.5–3.0
**Marital status**														
Single or widow or widower	12	71.0	5	29.0	0.051	1.0	-	12	71.0	5	30.0	0.092	1	-
Married	37	45.0	46	55.0	-	3.0	1.0–9.0	40	48.0	43	52.0	-	3.0	1.0–8.0
**Age group (years old)**														
20–40	17	61.0	11	39.0	0.263	1.0	-	18	64.0	10	36.0	0.238	1.0	-
>50	18	49.0	19	51.0	-	2.0	1.0–4.0	19	51.0	18	49.0	-	2.0	1.0–5.0
41–50	14	40.0	21	60.0	-	2.0	1.0–6.0	15	43.0	20	57.0	-	2.0	1.0–7.0
**Final education**														
Graduated from junior high school or below	20	61.0	13	39.0	0.103	1.0	-	18	54.0	15	45.0	0.721	1.0	-
Graduated from senior high school or above	29	43.0	38	57.0	-	2.0	1.0–5.0	34	51.0	33	49.0	-	1.0	0.5–3.0
**Working status**														
Irregularly work	24	54.0	20	45.0	0.462	1.0	-	27	62.0	17	38.0	0.189	1.0	-
Regularly work	17	49.0	18	51.0	-	1.0	0.5–3.0	17	49.0	18	51.0	-	2.0	1.0–4.0
Staff of *RW* or *RT* or *cadres* or comm. leaders	8	38.0	13	62.0	-	2.0	0.7–6.0	8	38.0	13	61.0	-	3.0	1.0–7.0
**Live in the same house with**														
Husband or wife or children or other family	24	58.0	17	41.0	0.112	1.0	-	22	54.0	19	46.0	0.782	1.0	-
Husband or wife or children or other family	25	42.0	34	58.0	-	2.0	1.0–4.0	30	51.0	29	49.0	-	1.0	0.5–2.0
**Number of owned children (person)**														
> 2	21	58.0	15	42.0	0.161	1.0	-	15	42.0	21	58.0	0.121	1.0	-
1–2	28	44.0	36	56.0	-	2.0	1.0–4.0	37	58.0	27	42.0	-	0.5	0.2–1.0
**Monthly income (IDR)**														
1-3 million	31	52.0	29	48.0	0.514	1.0	-	34	57.0	26	43.0	0.253	1.0	-
> 3 million	18	45.0	22	55.0	-	1.0	1.0–3.0	18	45.0	22	55.0	-	2.0	1.0–4.0
**Knowledge on KSB**														
Poor	27	64.0	15	36.0	0.009	1.0	-	32	76.0	10	24.0	0.001	1.0	-
Good	22	38.0	36	62.1	-	3.0	1.0–7.0	20	34.0	38	65.0	-	6.0	2.0–15.0
**Knowledge on natural disaster and flood**														
Poor	33	60.0	22	40.0	0.015	1.0	-	42	76.0	13	24.0	0.001	1.0	-
Good	16	36.0	29	64.0	-	3.0	1.0–6.0	10	22.0	35	78.0	-	11.0	4.0–29.0
**Flood early warning system**														
Poor	28	72.0	11	28.0	0.001	1.0	-	29	74.0	10	26.0	0.001	1.0	-
Good	21	34.0	40	66.0	-	5.0	2.0–12.0	23	38.0	38	62.0	-	5.0	2.0–12.0
**Emergency response plan**														
Poor	25	51.0	24	49.0	0.692	1.0	-	32	65.0	17	35.0	0.009	1.0	-
Good	24	47.0	27	53.0	-	1.0	0.5–3.0	20	39.0	31	61.0	-	3.0	1.0–7.0

**TABLE 8b:** Differences in flood management practices based on socio-demographic characteristics, *Kampung Siaga Bencana* knowledge, disaster knowledge, flood EWS, a demergency response plans for heat cadres and comm. leaders.

Variable	After flood				*p*	OR	95% CI OR	Flood management				*p*	OR	95% CI OR
	Poor		Good					Poor		Good				
	*n*	%	*n*	%				*n*	%	*n*	%			
**Position in the community**														
Chairman or staff of *RT* or *RW*	24	63.0	14	37.0	0.611	1.0	-	22	58.0	16	42.0	0.325	1.0	-
Youth organization or comm. leaders	12	52.0	11	48.0	-	2.0	1.0–4.0	12	52.0	11	48.0	-	1.0	0.4–4.0
Health cadres	25	64.0	14	36.0	-	1.0	1.0–2.0	16	41.0	23	59.0	-	2.0	1.0–5.0
**Sex**							-							
Male	31	62.0	19	38.0	0.838	1.0		28	56.0	22	44.0	0.230	1.0	-
Female	30	60.0	20	40.0	-	1.0	0.5–2.0	22	44.0	28	56.0	-	2.0	1.0–4.0
**Marital status**														
Single or widow or widower	14	82.0	3	18.0	0.048	1.0	-	12	71.0	5	29.0	0.062	1.0	-
Married	47	53.0	36	43.0	-	4.0	1.0–13.0	38	46.0	45	54.0	-	3.0	1.0–9.0
**Age group (years old)**														
20–40	20	71.0	8	29.0	0.266	1.0	-	18	64.0	10	36.0	0.156	1.0	-
>50	23	62.0	14	38.0	-	1.0	1.0.–4.0	18	49.0	19	51.0	-	2.0	1.0–5.0
41–50	18	51.0	17	49.0	-	2.0	1.0–7.0	14	40.0	21	60.0	-	3.0	1.0–7.0
**Final education**														
Graduated from junior high school or below	23	70.0	10	30.0	0.211	1.0	-	20	61.0	13	39.0	0.137	1.0	-
Graduated from senior high school or above	38	57.0	29	43.0	-	2.0	1.0–4.0	30	45.0	37	55.0	-	2.0	1.0–4.0
**Working status**														
Irregularly work	28	64.0	16	36.0	0.872	1.0	-	23	52.0	21	48.0	0.208	1.0	-
Regularly work	21	60.0	14	40.0	-	1.0	0.5–3.0	20	57.0	15	43.0	-	1.0	0.3–2.0
Staff of *RW or RT or cadres or* comm. leaders	12	57.0	9	43.0	-	1.0	0.5–4.0	7	33.0	14	67.0	-	2.0	1.0–6.0
**Live in the same house with**														
Husband/wife/children/other family	25	61.0	16	39.0	0.997	1.0	-	22	54.0	19	46.0	0.542	1.0	-
Husband or wife or children or other family	36	61.0	23	39.0	-	1.0	0.4–2.0	28	47.0	31	52.0	-	1.0	1.0–3.0
**Number of owned children (person)**														
> 2	21	58.0	15	42.0	0.682	1.0	-	19	53.0	17	47.0	0.677	1.0	-
1–2	40	62.0	24	37.0	-	1.0	0.4–2.0	31	48.0	33	52.0	-	1.0	0.5–3.0
**Monthly income (IDR)**														
1-3 million	38	63.0	22	37.0	0.558	1.0	-	30	50.0	30	50.0	1.000	1.0	-
> 3 million	23	57.0	17	42.0	-	1.0	1.0–3.0	20	50.0	20	50.0	-	1.0	0.5–2.0
**Knowledge on KSB**														
Poor	32	76.0	10	24.0	0.008	1.0	-	30	71.0	12	29.0	0.001	1.0	-
Good	29	50.0	29	50.0	-	3.0	1.0–8.0	20	34.0	38	65.0	-	5.0	2.0–11.0
**Knowledge on natural disaster and flood**														
Poor	45	82.0	10	18.0	0.001	1.0	-	38	69.0	17	31.0	0.001	1.0	-
Good	16	36.0	29	64.0	-	8.0	3.0–20.0	12	27.0	33	73.0	-	6.0	3.0–15.0
**Flood early warning system**														
Poor	31	79.0	8	20.0	0.002	1.0	-	26	67.0	13	33.0	0.008	1.0	-
Good	30	49.0	31	51.0	-	4.0	2.0–10.0	24	39.0	37	61.0	-	3.0	1.0–7.0
**Emergency response plan**														
Poor	34	69.0	15	31.0	0.092	1.0	-	29	59.0	20	41.0	0.072	1.0	-
Good	27	53.9	24	47.0	-	2.0	1.0–5.0	21	41.0	30	59.0	-	2.0	1.0–5.0

**TABLE 9:** Multivariate analysis of the dominant variable of flood management practices among health cadres and community leaders.

Variable	Logistic coefficients (B)	Standard error (s.e.)	Wald	db	*p*	OR	95% CI OR	
							BB	BA
**Before flood**								
Position at community	-	-	6.62	2	0.037	-	-	-
Position at community (youth organization, community leaders, vs. staff of RW/RT)	0.74	0.65	1.32	1	0.251	2.10	0.59	7.46
Position at community (health cadre vs. staff of RW/RT)	1.43	0.56	6.61	1	0.010	4.18	1.40	12.43
Marital status (married vs. single or widow or widower)	1.35	0.69	3.86	1	0.050	3.86	1.00	14.85
Final education (graduated from senior high school or above vs. graduated from junior high school or below)	1.38	0.54	6.48	1	0.011	3.98	1.37	11.52
Flood early warning system (good vs. poor)	1.84	0.51	13.10	1	0.000	6.33	2.33	17.18
Constant	−3.90	1.02	14.58	1	0.000	0.02	-	-
*-2LL = 110.0, Cox and Snell R Square = 0.25, Nagelkerke R Square = 0.33, Omnibus Test Model = 28.6, p = 0.001*								
**During flood**								
Knowledge of health cadres or community leaders on KSB (good vs. poor)	1.42	0.53	7.02	1	0.008	4.12	1.45	11.74
Knowledge of health cadres or community leaders on natural disaster and flood (good vs. poor)	2.10	0.51	16.86	1	0.000	8.15	2.99	22.19
Emergency response plan (good vs. poor)	1.05	0.51	4.16	1	0.041	2.86	1.04	7.83
Constant	−2.45	0.58	17.60	1	0.000	0.09	-	-
*-2LL = 96.2. Cox and Snell R Square = 0.36, Nagelkerke R Square = 0.46, Omnibus Test Model = 42.2, p = 0.001*								
**After flood**								
Knowledge of health cadres or community leaders on natural disaster and flood (good vs. poor)	2.10	0.47	20.09	1	0.000	8.16	3.26	20.42
Constant	−1.50	0.35	18.51	1	0.000	0.22	-	-
*-2LL = 110.7. Cox and Snell R Square = 0.21, Nagelkerke R Square = 0.28, Omnibus Test Model = 23.0, p = 0.001*								
**Flood management**								
Knowledge of health cadres or community leaders on KSB (good vs. poor)	1.16	0.47	5.97	1	0.015	3.18	1.26	8.03
Knowledge of health cadres or community leaders on natural disaster and flood (good vs. poor)	1.51	0.47	10.39	1	0.001	4.52	1.81	11.30
Constant	−1.35	0.39	11.94	1	0.001	0.26	-	-
*-2LL = 114.2, Cox and Snell R Square = 0.22, Nagelkerke R Square = 0.29, Omnibus Test Model = 24.5, p = 0.001*								

CI, confidence interval; OR, odd ratio.

## Discussion

Families with disaster-vulnerable groups had a low level of knowledge about KSB, inadequate flood warning system and emergency response plan parameters, and inadequate flood management practices compared to *posyandu* cadres and community leaders; yet, they need better family support. Health cadres and community leaders are community groups who are tasked with supporting the health programs at the *RT* and *RW* levels. A community leader is a person who is a role model for the community in a particular region. Cadres are agents of change in health and nutrition programs at the *posyandu* for under-five children and *posbindu* for older people (Yulidar et al. 2020). Health cadres and community leaders are crucial in improving the preparedness, emergency response, and post-flood practices of communities living in flood-prone areas to enable them to act as quickly and effectively as possible. Therefore, these two groups must know more about KSB, natural hazards, and floods than the general population. All locations in this study already have KSB established by the Ministry of Social Affairs of the Republic of Indonesia based on the Regulation of the Minister of Social Affairs of the Republic of Indonesia No. 128 of 2011 (Audit Board of the Republic of Indonesia (BPK) 2011). The purpose of establishing KSB is to create public understanding and awareness of disaster hazards; establish a community-based disaster preparedness network and strengthen social interaction of community groups; organize trained disaster preparedness communities; ensure the implementation of sustainable community-based disaster preparedness; and optimize potentials and resources for disaster management. Disaster Alert Villages are established in disaster-prone areas, including those prone to floods, and have a high level of community participation. Almost all respondents from the cadre and community leader group joined the KSB so that they know KSB, know of natural hazards and floods, flood early warning systems, and have good emergency response plans (BPK 2011).

Family support in flood management practices is already good in terms of providing information on equipment and actions that need to be taken before the event of a flood, reminding older people constantly to activate their communication devices to make it easier for them to contact others during emergency conditions, and preparing vehicles to evacuate family groups who are vulnerable to flood to a safe location in an emergency. Family support is an attitude, an act of acceptance towards family groups, in the form of giving informational, assessment, instrumental, and emotional support. There are four types of family support: informational, physiological, instrumental, and emotional support (Friedman 2010). Informational, instrumental, and emotional supports are provided in this study. This is in line with earlier studies conducted on the effect of family support on older people’s preparedness (Djaafar et al. 2021; Nurhidayati 2018). Families and groups vulnerable to flood are the main stakeholders in disaster management.

**FIGURE 6:**
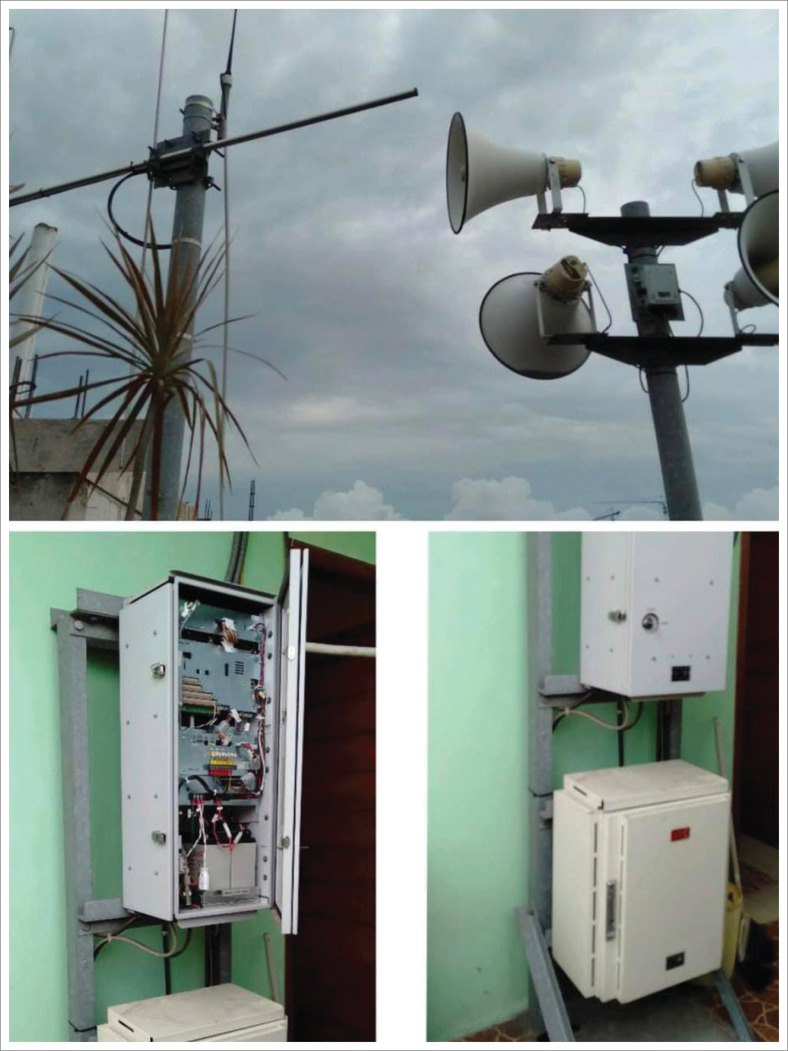
Disaster early warning system (DEWS) for a flood at Rawa Buaya Urban Village.

Early warning systems are crucial in disaster management, consisting of knowledge of risk, monitoring and warning services, dissemination and communication, and countermeasure capabilities. Flood early warning systems are the most critical variable that determines the practice of families with groups vulnerable to floods, such as children, pregnant women, nursing mothers, persons with disabilities, and older people, before and during flood emergency response. Respondents use various flood early warning systems, including WhatsApp Group (WAG), mosque/*musholla* speaker announcements, sirens from village offices, and flood detection early warning system (DEWS) tools (Figure 6). The DEWS tool is a remote loudspeaker/speaker in the form of a pole with loudspeakers on all four sides pointing in various directions to announce information to the community in flood-prone areas. This tool is activated when the river floodgates begin to enter alert level 3, alert level 2, and alert level 1. The BPBD of DKI Jakarta Province activates the DEWS tool directly using VHF digital radio technology (Aji 2020; Wibowo 2023). In Rawa Buaya Village, local people have developed a simple way of early detection of floods through marks on concrete pillars of the railway bridge. Green, yellow, and red marks are water level markers, with yellow indicating alert and red indicating danger or flood alert. Early warning systems are a crucial component of disaster management. They encompass a range of activities, including risk assessment, monitoring and warning services, communication and dissemination, and countermeasure capabilities (International Strategy for Disaster Reduction [ISDR] 2006).

Early warning of the flood is a significant factor in reducing flood risk because the community can respond accordingly to conduct rescues, avoid casualties, and reduce the impact of the disaster. Early flood warnings help warn residents of the potential for floods through communication devices or by directly using sirens and warning lights so that rescue measures can be taken immediately. A complete, adequate, and community-centered flood early warning requires four elements of knowledge of disaster risk, monitoring, and warning services through the establishment of hazard monitoring and early warning services, dissemination and communication of risk and early warning information, and community capabilities (Sagay & Pangemanan 2023). The findings of this study demonstrated that the majority of respondents when they hear the sound of sirens or *kentongan* (a traditional slit drum usually used as a communication tool in the village) signalling the possibility of flood, will immediately evacuate to a safe location. In addition, respondents immediately helped the flood-vulnerable groups reach safe meeting points.

Knowledge of natural hazards and floods is the primary determinant of flood management practices by families with groups vulnerable to flood. The same applies to the *posyandu cadres* and community leaders who participated in this study. Experience provides knowledge about floods hitting the area and will affect the attitude and awareness of the community to be ready to anticipate floods. The community’s knowledge of disasters can affect their awareness and preparedness when flooding strikes flood-prone areas (Kim & Kim 2022; Noor et al. 2022; Oktari et al., 2020; Shanableh et al. 2023). This study’s findings align with several studies conducted on community knowledge and flood preparedness (Nekada, Herawati & Rahil 2023; Yatnikasari, Asnan & Agustuna 2021). A community’s knowledge about disasters will affect household attitudes and awareness to be ready and prepared to anticipate disasters to minimize the follow-up impacts of disasters (LIPI-UNESCO/ISDR 2006). The emergency response plan also plays a role in flood management practices as shown in this study. Emergency response plans include plans to respond to emergencies, evacuate, provide first aid, rescue, safety and security, fulfilment of basic needs, equipment and tools, essential facilities, and simulation or exercises (Firmansyah 2014; Taryana, Mahmudi & Bekti 2022). This study’s findings are supported by a survey carried out in Pacitan District stating that an emergency response plan is a learning process to improve the community’s ability to deal with disasters (Faturahman 2018).

A flood preparedness framework is needed to assess the extent of community preparedness in an area facing natural hazards. The first parameter is knowledge, which is the main factor and is critical to readiness, including the practice of being ready and prepared before a flood, especially for people living in flood-prone areas. The second parameter is an emergency response plan linked to self-evacuation to safe meeting points, evacuation routes, first aid, and self-rescue during flooding. Flood early warning systems include warning signs and distribution of information about the occurrence of disasters. The community can significantly take appropriate actions through disaster warnings to reduce casualties. For this reason, training and simulation are needed to familiarize people with what to do when hearing warnings and where and how to save themselves within a specific time frame according to where the people are during the disaster warning. Human resource mobilization is performed through family and community participation in flood preparedness training, and first aid training, among others (Deni 2008).

Health cadres and community leaders already have good flood management practices before, during, and after floods. This condition is based on sound knowledge of natural hazards and floods and KSB, along with the presence of flood early warning systems and emergency response plans. Based on these three disaster preparedness parameters, it is necessary to establish flood KSBs in the five study locations that have already become Disaster Alert Villages. The strengthening of KSB is facilitated by family support for the family groups who belong to the flood-vulnerable groups, establishing a Disaster Alert Village, and the willingness and readiness of health cadres and community leaders to become KSB. For families with groups vulnerable to flood, efforts need to be made to increase preparedness, including providing disaster knowledge education on flood mitigation and preparedness strategies, emergency response plans, and resource mobilization. To support KSB skills, it is necessary to do simulations or exercises and management training on floods and other disasters with BPBD DKI Jakarta Province, the Ministry of Social Affairs, non-governmental organizations (NGOs), and related parties.

The study has some limitations. Firstly, the cross-sectional design of this study cannot assess the causal and influence relationships between independent variables, such as socio-demographic characteristics, family support, disaster knowledge, flood early warning system, and emergency response plan, with the dependent variable of flood management practices. Secondly, the flood-vulnerable groups studied are not specific to one type of vulnerable group, such as children, pregnant women, nursing mothers, older people, and persons with disabilities. As a result, the identified flood management practices (before, during, and after) are general. Thirdly, the study locations were chosen by purposive sampling based on data from BPBD DKI Jakarta province that cannot be generalized to the DKI Jakarta Provincial level. Fourthly, this study uses a quantitative approach with quantitative methods. It is necessary to use a mixed quantitative and qualitative method to explore why and how both types of respondents (families and prospective KSB) perform good flood management practices.

## Conclusion

Generally, the four community preparedness parameters, namely disaster knowledge, emergency response plan, flood early warning system, and human resource mobilization still need to be improved. Only family support is found to be good in this respondent group. The KSB candidates, including health cadres and community leaders, have four suitable preparedness parameters, especially in disaster knowledge, which plays a significant role in flood management practices. Disaster education from KSB in simulations, exercise, and training needs to be provided more intensively to families with groups vulnerable to flood to improve preparedness parameters. The role and tasks of the Disaster Preparedness Village need to be intensified to strengthen the role of KSB as an agent of change to improve community preparedness in flood management.
